# A mass balance study of [^14^C]SHR6390 (dalpiciclib), a selective and potent CDK4/6 inhibitor in humans

**DOI:** 10.3389/fphar.2023.1116073

**Published:** 2023-03-31

**Authors:** Hua Zhang, Shu Yan, Yan Zhan, Sheng Ma, Yicong Bian, Shaorong Li, Junjun Tian, Guangze Li, Dafang Zhong, Xingxing Diao, Liyan Miao

**Affiliations:** ^1^ Department of Clinical Pharmacology, The First Affiliated Hospital of Soochow University, Suzhou, China; ^2^ Institute for Interdisciplinary Drug Research and Translational Sciences, Soochow University, Suzhou, China; ^3^ Shanghai Institute of Materia Medica, Chinese Academy of Sciences, Shanghai, China; ^4^ Jiangsu Hengrui Medicine Co., Ltd., Lianyungang, Jiangsu, China

**Keywords:** SHR6390, [^14^C]SHR6390, radioactivity, drug metabolism, CDK, pharmacokinetics

## Abstract

SHR6390 (dalpiciclib) is a selective and effective cyclin-dependent kinase (CDK) 4/6 inhibitor and an effective cancer therapeutic agent. On 31 December 2021, the new drug application was approved by National Medical Product Administration (NMPA). The metabolism, mass balance, and pharmacokinetics of SHR6390 in 6 healthy Chinese male subjects after a single oral dose of 150 mg [^14^C]SHR6390 (150 µCi) in this research. The *Tmax* of SHR6390 was 3.00 h. In plasma, the *t*_1/2_ of SHR6390 and its relative components was approximately 17.50 h. The radioactivity B/P (blood-to-plasma) AUC_0-t_ ratio was 1.81, indicating the preferential distribution of drug-related substances in blood cells. At 312 h after administration, the average cumulative excretion of radioactivity was 94.63% of the dose, including 22.69% in urine and 71.93% in stool. Thirteen metabolites were identified. In plasma, because of the low level of radioactivity, only SHR6390 was detected in pooled AUC_0-24 h_ plasma. Stool SHR6390 was the main component in urine and stool. Five metabolites were identified in urine, and 12 metabolites were identified in stool. Overall, faecal clearance is the main method of excretion.

## 1 Introduction

The disorder of cell division, which leads to abnormal cell proliferation, is one of the key signs of cancer. In cancer treatment, the target of blocking cell division is a very important research goal. Cell cycle usually refers to the stage in which cells pass through a predetermined number of stages under the control of a complex network of regulators (Hartwell et al., 1974). The cell cycle consists of several different stages (Malumbres and Barbacid, 2001). The initiation of cell cycle requires the induction of cyclin and cyclin dependent kinases (CDKs) expression through growth factors, estrogen and other mitogenic stimuli (de Dueñas et al., 2018). Breast cancer is associated with the imbalance of D-cyclin dependent kinase 4/6-retinoblastoma (cyclin D-CDK4/6-retinoblastoma) pathway (Arnold and Papanikolaou, 2005; Cancer Genome Atlas Network, 2012; Witkiewicz and Knudsen, 2014). Class D cyclins (D1, D2 and D3) are regulators of CDK4 and CDK6 kinases, and together form active complexes (Weinberg, 1995). Among them, CDK4/6 manages the process of cell cycle through reversible binding with cyclin D1. At the early stage of G1, active CDK4 and CDK6 phosphorylate retinoblastoma (RB) protein (a tumor inhibitor), leading to partial release of E2F transcription factor, and then promoting the transcription of downstream genes required to enter S phase through G1 restriction point (Morgan, 1997; Lundberg and Weinberg, 1998). P16 is an endogenous CDK4 inhibitor, which plays a role in reducing cell cycle and is often expressed as loss in malignant tumors (Bartkova et al., 1996). A key feature of tumorigenesis is the uncontrolled proliferation of cells, which is due to the disorder of cell cycle regulation. Therefore, cyclin D1-CDK4/6-RB pathway is a good target for anticancer drugs (Long et al., 2019).

First-generation CDK inhibitors are non-selective universal CDK blockers with limited antitumour activity and obvious toxicity (Shapiro, 2006). More recently, in the treatment of metastatic breast cancer, selective small molecule CDK4/6 inhibitors have also become increasingly effective, such as palbociclib, ribociclib and abemaciclib, which have been developed in metastatic luminal breast cancer (Cadoo et al., 2014). Three CDK4/6 inhibitors, Palbicilib, Ribocib and Abemacilib, which were previously marketed. The chemical structures of Palbicilib and ribocib are similar and have good selectivity. Abemachilib is different from them in structure. Its structure can inhibit other kinases, such as CDK9 (Gelbert et al., 2014). In addition, these CDK4/6 inhibitors show differences in terms of toxicity, so they correspond to different administration schemes. Palbociclib and ribociclib induce bone marrow suppression, which is usually administered for 1 week to restore the neutrophil count in patients, whereas abemaciclib is dosed continuously and elicits fatigue and diarrhoea as more relevant dose-limiting toxicities (Asghar et al., 2015).

SHR6390 (dalpiciclib) is a selective and effective cyclin-dependent kinase (CDK) 4/6 inhibitor and an effective cancer therapeutic agent. On 31 December 2021, the new drug application was approved by NMPA (National Medical Product Administration). SHR6390 exhibited potent antiproliferative activity against a wide range of human RB- positive tumor cells, and exclusively induced G1 arrest as well as cellular senescence, with a concomitant reduction in the levels of Ser780-phosphorylated RB protein. Although many research results on SHR6390 have been published, there are still many problems of concern that have not been solved or disclosed (Wang et al., 2017; Long et al., 2019; Chen et al., 2020; Zhang et al., 2021). To date, there are no data to evaluate its overall metabolism in humans. It is very important to understand the metabolism of SHR6390 through radioactive substances so as to evaluate its safety in the future (Robison and Jacobs, 2009; Penner et al., 2012; Prakash et al., 2019). This research can also help guide the clinical evaluation of SHR6390 in the future and help to select the appropriate dose. The use of radioactive tracers in pharmacokinetic studies enables us to better understand the excretion pathway and metabolism of drugs (Murai et al., 2014; Lappin, 2015; Meng et al., 2019; Yamada et al., 2019; Tian et al., 2021; Zheng et al., 2021). Therefore, in this study, the pharmacokinetics, biotransformation pathway and mass balance of [^14^C]SHR6390 in humans were investigated.

## 2 Materials and methods

### 2.1 Chemicals and reagents

SHR6390 (purity 99.50%) was provided by Jiangsu Hengrui Medicine Co., Ltd. (Lianyungang, China). [^14^C]SHR6390 (150 μCi, purity 98.62%) and SHR6390 (150 mg) were dissolved in 5% carboxymethylcellulose sodium (CMC-Na, purchased from Aladdin, Shanghai, China) and stored at approximately −20°C. For other reagent information, please refer to another article in our group (Zheng et al., 2021).

### 2.2 Instruments

High-resolution mass spectrometry (HR-MS) and HR-MS^2^ acquisition are currently widely used in the field of metabolite identification, while background subtraction and mass loss filtering techniques have promoted the development of metabolite identification (Zhang et al., 2008; Zhang et al., 2009; Ming Yao et al., 2020). In this study, data are collected through the XCalibur and Laura systems. A Vanquish Ultra High Performance Liquid Chromatography (UHPLC) system was used to carry out detection with a Q Executive Plus mass spectrometer (Thermo, MA, United States). The system setting are shown in Table 1. The mass spectrum data were analysed using Compound Discoverer software (Themo).

**TABLE 1:** UHPLC-HRMS setting.

UHPLC condition		
Colume	ACQUITY UPLC HSS T3 (100 mm × 2.1 mm, 1.8 µm, Waters, United States)	
Phase A	5 mM ammonium acetate aqueous solution	
Phase B	Acetonitrile	
UV detection	254 nm	
Gradient elution	time (min)	B (%)
	0	10
	2	10
	24	30
	26	95
	28.8	95
	28.9	10
	35	10
MS condition		
Source	ESI	
Mode	Positive	
Scan range	100–1,000 Da	
Sheath gas	45 L/min	
Aux gas	10 L/min	
Capillary temperature	320°C	
Capillary voltage	3.5 kV	

### 2.3 Design, subjects and sample collection

The clinical trial (No. CTR20230830) was an open-label, single-center, single-dose trial conducted at the First Affiliated Hospital of Soochow University (Suzhou, China). This study was conducted in accordance with the ethical principles required by the Helsinki Declaration and approved by the Hospital Ethics Committee (2020. No.151). Six healthy Chinese male subjects were recruited between 18 and 45 years old with a body mass index between 19 and 26 kg/m^2^. All subjects signed a written ICF (informed consent form) before the start of the study. Plasma samples were collected from pre-dose to 144 h after dose. Urine and stool samples were collected pre-dose to 312 h after dose administration. The standards of subject out were the following three criteria: The cumulative excretion radioactivity exceeded 80% of the dose radioactivity; the radioactivity excreted was less than 1% of the radioactivity administration over a 24 h period on two consecutive days; and the measured radioactivity in the collected plasma was 3 times lower than that of the pre-dose (Bian et al., 2021). Fasting for at least 10 h and then water deprivation for 1 h, each subject was given a single oral dose of 150 mg [^14^C]SHR6390 (150 µCi) suspension. Rinse the dosing bottle with warm water and give it to the subjects. The total volume of drug preparation and lotion did not exceed 240 mL. After taking the medicine, the subjects fasted for 4 h and refrained from water for 1 h after dosing. Twenty millilitres of whole blood was collected before administration and 2, 6, 10, 24 and 48 h after administration. In 20 mL of whole blood, 1.6 mL was used for the test, and 0.4 mL was placed in the backup tube. Centrifuge (3,500 rpm, 5 min, 4°C) 10 mL of whole blood to produce plasma. The volume of plasma in one of the two tubes was 3.2 mL, and the remaining plasma was put into the backup tube. The remaining 8 mL whole blood was centrifuged (3,500 rpm, 5 min, 4°C) to produce plasma for metabolic study. Meanwhile, 10 mL whole blood was collected at 0.5, 1, 3, 4, 8, 72, 96, 120, and 144 h. The plasma required for detection is obtained by whole blood centrifugation. In addition to collecting urine samples before administration and 0–4 h, 4–8 h, 8–12 h and 12–24 h after administration, urine samples will be collected every 24 h in the following collection periods. Collection of fecal samples, except before administration, shall be conducted at 24-h intervals after administration. Plasma samples were stored at −80°C, and urine and stool samples were stored at −20°C until analysis.

### 2.4 Radioactivity

The radioactivity of urine and plasma was detected by liquid scintillation counter (LSC) (Tri-Carb 3110 TR, PerkinElmer, MA, United States). Two times the weight of acetonitrile-water (1:1, *v*: *v*) was added to the stool and homogenized. Blood and stool homogenate were weighed and burned in a biological oxidizer (OX-501, Harvey, NY, United States). Then, the CO_2_ with 14C labled was trapped in the liquid scintillation cocktail (RDC, NJ, United States) and detected by LSC.

### 2.5 Radioprofiling

#### 2.5.1 Recovery

The total extraction recovery was 119%, 84.69% and 116.64% in plasma, urine and feces, respectively. The colume recovery was 92.21%, 100.14%, 99.13% in plasma, urine and feces, respectively. The recovery improved the method of extraction and LC-MS was suitable.

#### 2.5.2 Plasma

According to the AUC principle, the plasma of 6 subjects from 0–24 h was pooled (Hop et al., 1998). The plasma sample (15 mL) after pooled was added 15 mL methanol and 15 mL acetonitrile and centrifugation (3,500 rpm, 10 min, 4°C). Extract the centrifuged solid with 7.5 mL water and 22.5 mL methanol acetonitrile (50:50, *v*: *v*). The first two extracted supernatants were combined and concentrated. The concentration of the substance is at 200 μL Acetonitrile water (20:80, *v*: *v*) was dissolved again and centrifuged again (3,600 rpm, 10 min, 4°C). Part of the supernatant was injected into the UHPLC-FC (Fraction collector) system (Thermo). The eluent from the UHPLC was collected into the Deepwell LumaPlate 96 (PerkinElmer) at the rate of 10 s per well within minutes. The total collection time is 35 min. The plates were dried by Integrated SpeedVac (Thermo), and the radiation value of each well were detected by a microplate reader (Sense Beta Hidex, Finland). Data were reconstructed to radio-chromatogram by Laura software (Lablogic, United Kingdom) to give the radio profiling of plasma.

#### 2.5.3 Urine

According to the principle of equal volume, the urine samples of 6 subjects from 0 to 120 h were merged. The combined samples were centrifuged, concentrated by N_2_ and dissolved in a 200 μL mixture of 40 mL acetonitrile and 160 mL-water, and 120 μL was injected into the UHPLC-FC system (Thermo). Other operation steps are the same as those of plasma.

#### 2.5.4 Stool

According to the principle of equal proportion weight, the fecal homogenates of 6 subjects from 0 to 168 h were combined. Add 6 mL methanol acetonitrile (50:50, v: v) to the combined fecal homogenate (2 g) sample. The mixture was then whirled (1 min) and centrifuged6 (3,500 rpm, 10 min, 4°C). Transfer the supernatant into a clean tube, and then extract the extracted solid again with 2 mL water and 2 mL methanol and 2 mL acetonitrile. The two supernatants were combined, concentrated by N_2_ at 25°C and dissolved in acetonitrile-water (20: 80, v: v) of 200 μL, and 60 μL was injected into the UHPLC-FC system (Thermo). Other operation steps are the same as those of plasma.

### 2.6 Metabolite identification

The MS signal of the metabolites were obtained through UHPLC-HRMS, and the metabolic pathway of the metabolite was speculated. Through the MSMS spectrum obtained, the structure of the metabolite was identified by comparing the mass spectrum fragment with the mass spectrum fragment produced by the parent compound.

### 2.7 Pharmacokinetic analysis

The application software Phoenix WinNonlin (Version 7.0; Pharsight Corporation, Mountain View, CA) used a non-compartment model to calculate the parameters related to drug metabolism in this experiment. The related pharmacokinetic parameters for radioactivity are summarized in Table 2. By measuring the concentration of radioactive drugs in urine and stool, calculate the radioactive excretion rate (dose percentage) of each sample collected.

**TABLE 2:** Pharmacokinetic parameters of total radioactivity and SHR6390 in plasma after a single oral administration of [^14^C]SHR6390 to healthy volunteers [mean (s.d.)].

Parameter	Unit	^14^C plasma	SHR6390
*C*_max_	ng eq./mL	167 (19.1)	42.9 (10.4)
AUC_last_	ng eq./mL*h	1,670 (668)	1,150 (198)
AUC_inf_	ng eq./mL*h	3,930 (1,250)	1,250 (206)
*t*_1/2_	h	17.50 (7.92)	43.5 (7.77)
*T*_max_	h	3.17 (1.83)	3.00 (1.79)

## 3 Result

### 3.1 HR-MS analysis of SHR6390

**FIGURE 1:**
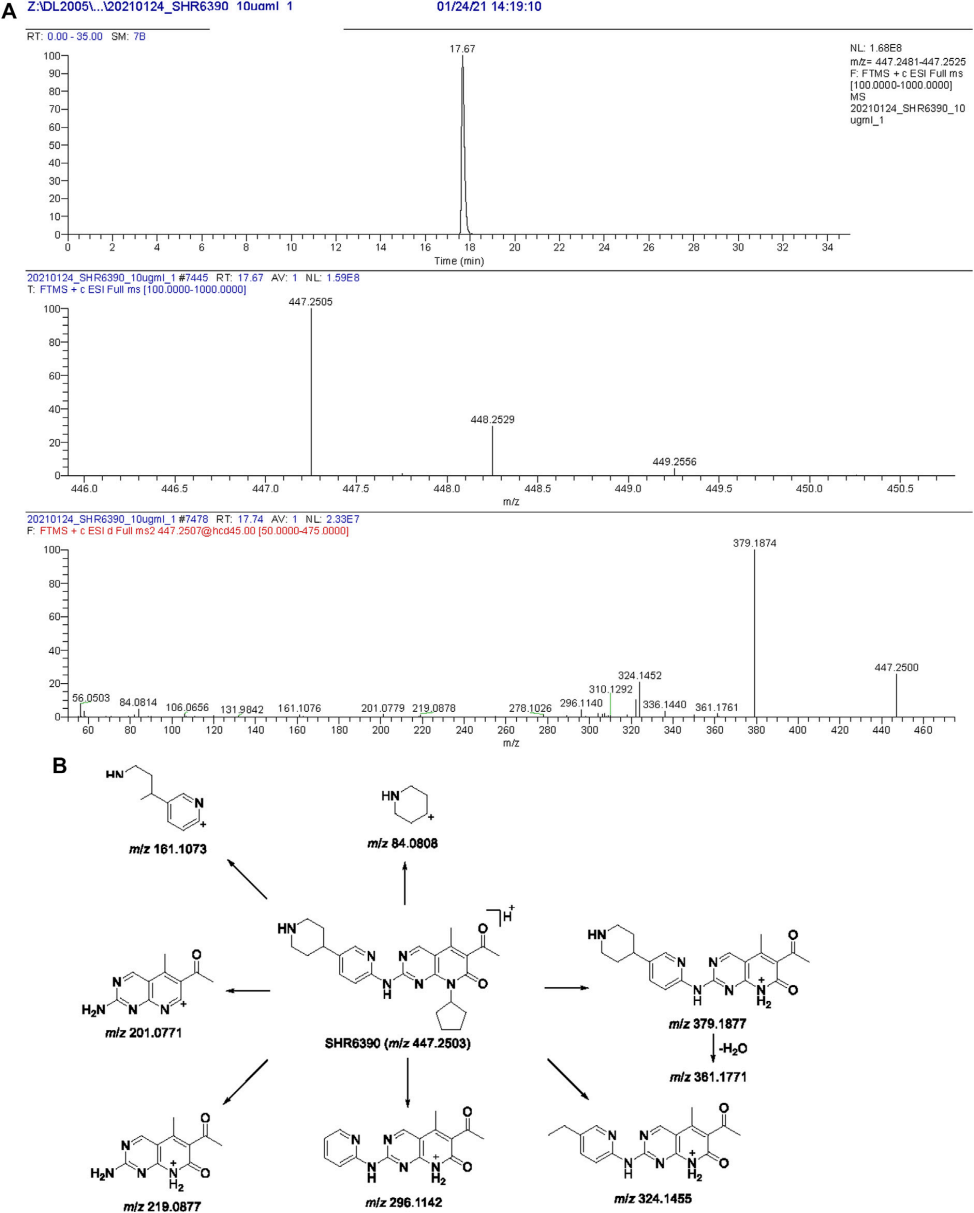
Extracted ion chromatograms, product ion spectra **(A)** and proposed fragmentation patterns of SHR6390 **(B)**.

Chromatographic and HR-MS fragmentation of SHR6390 was studied. The structural analysis of metabolites is based on the structural analysis of the parent drug. SHR6390, C_25_H_30_O_2_N_6_·C_2_H_6_O_4_S, with [M + H]^+^ at *m/z* 447.2503 eluted at 17.67 min and showed product ions at *m/z* 84.0808, 161.1073, 201.0771, 219.0877, 296.1142, 324.1455, 361.1771 and 379.1877 (Figures 1A, B). The base peak ion at *m/z* 379.1877 was generated by N-C cleavage of cyclopentane; further neutralization of H_2_O and C_3_H_5_N led to *m/z* 361.1771 and 324.1455, respectively. Based on the peak ion, *m/z* 379.1877 underwent further N-C cleavages, generating *m/z* 219.0877 and 201.0771.

### 3.2 Pharmacokinetics

The radioactivity concentration-time profiles, the *C*_max_ of radioactivity was 166 ng eq./mL. The mean AUC_last_ value was 1,670 ng eq./mL*h. The mean *T*_max_ and *t*_1/2_ were approximately 3.17 and 17.50 h, respectively. The radioactivity blood-to-plasma AUC_inf_ ratio (BPAR) of was 1.81. For SHR6390 in plasma, the mean C_max_ value of radioactivity was 42.9 ng/mL, and the mean AUC_last_ value was 1,150 ng/mL h. The mean AUC_inf_ values were 1,250 ng eq./mL*h. The mean T_max_ and *t*_1/2_ were approximately 3.00 and 43.5 h, respectively.

### 3.3 Mass balance

**FIGURE 2:**
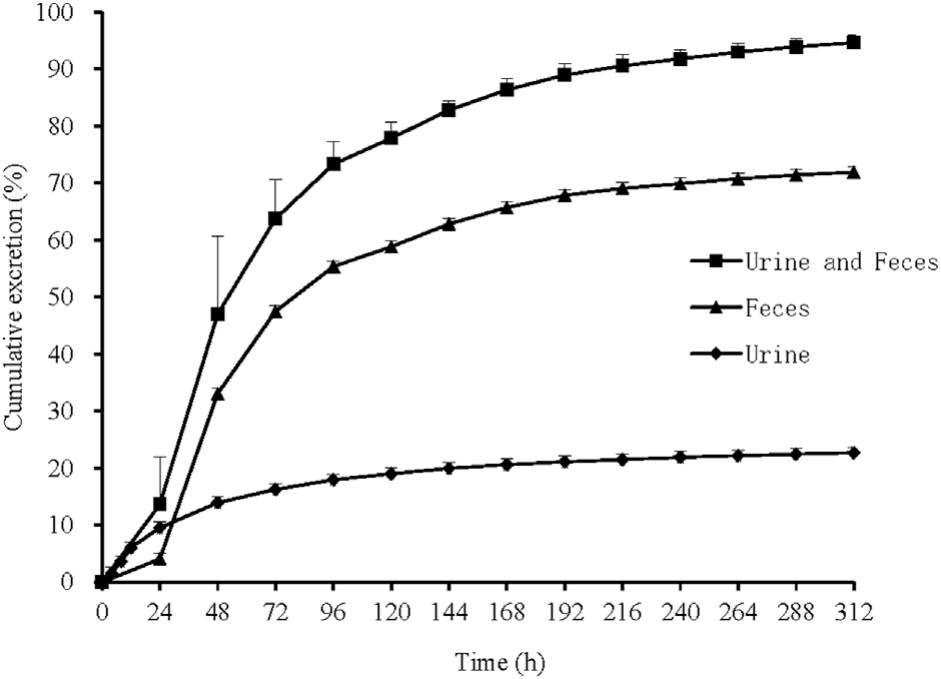
Mean cumulative excretion of total radioactivity in urine and feces following a single oral administration of [^14^C]SHR6390. Each point represents the mean ± S.D. of six subjects.

In 6 healthy Chinese male subjects after an oral dose of 150 mg [^14^C]SHR6390 (150 µCi), the recovery of total radioactivity was 94.63% (range 92.85%–96.34%). Stool excretion was the predominant route of elimination, accounting for 71.93% of the administered dose, while the mean urinary excretion was 22.69%. The total recovery in urine and stool in radioactive, 312 h after dosing was 94.63% (Figure 2).

### 3.4 Quantitative metabolite profiling

**FIGURE 3:**
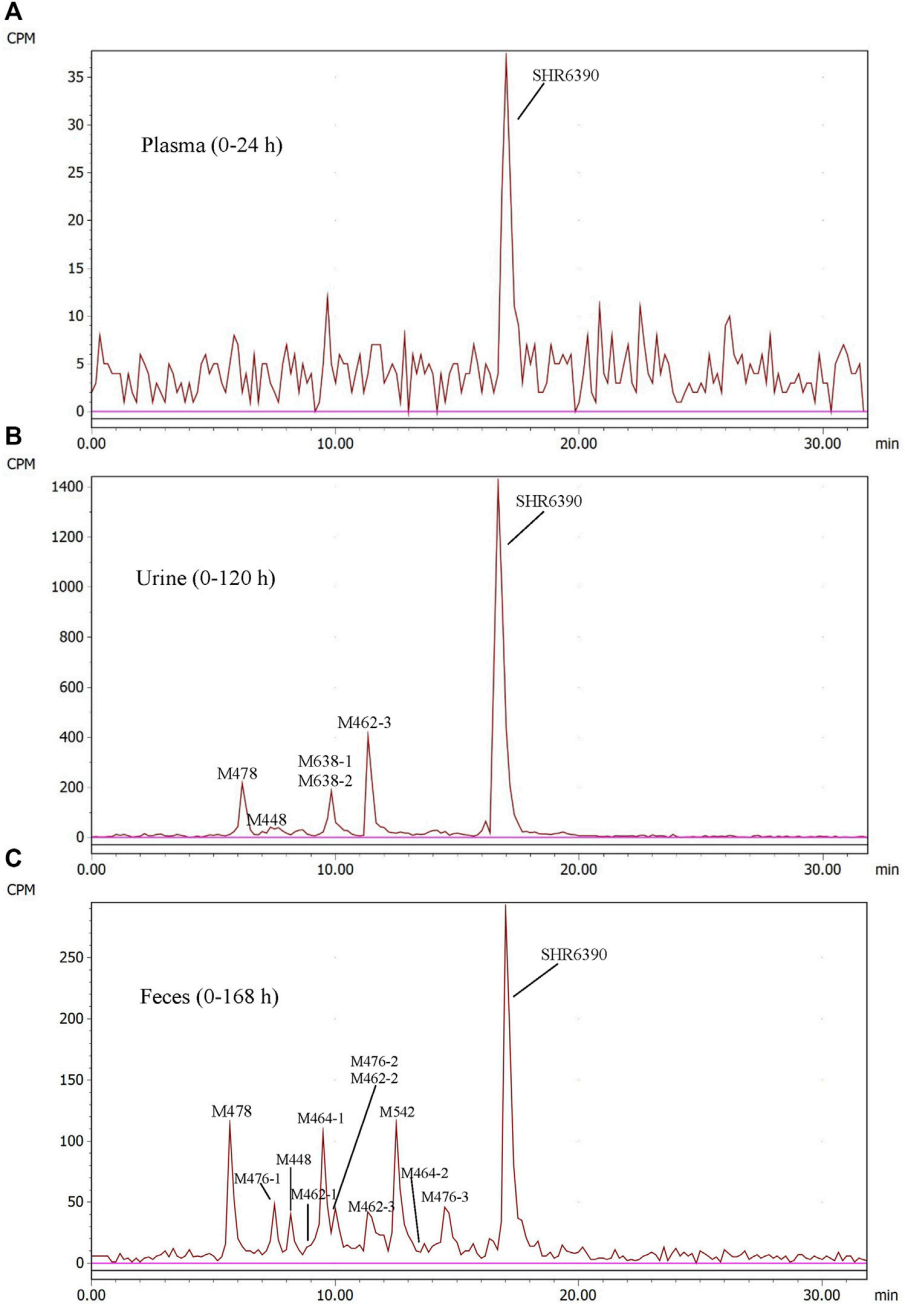
Representative radio-chromatograms of metabolites in human plasma (0–24 h) **(A)**, urine (0–120 h) **(B)**, and feces (0–168 h) **(C)** following oral administration of 150 mg [^14^C]SHR6390 (150 µCi).

Radio-chromatograms of each matrix are shown in Figure 3. Table 3 summarizes some characteristics of 13 metabolites, from which the structure identification of metabolites can also be confirmed. The naming rule of metabolites is ‘M + molecular weight.

**TABLE 3:** Information on SHR6390 metabolites detected in human plasma, urine, and stool.

ID	Metabolic pathway	Formula	Retention time (min)	[M + H]^+^ (determined)	Mass error (ppm)	Fragment ions
SHR6390	Parent	C_25_H_30_O_2_N_6_	16.67–17.00	447.2510	1.7	379.1847, 324.1452, 296.1140, 219.0878, 201.0779, 161.1076
M478	2[O]	C_25_H_30_O_4_N_6_	5.20–6.00	479.2410	1.8	411.1775, 393.1670,365.1721, 349.1770, 223.1552, 178.1339
M476-1	2[O]+[-2H]	C_25_H_28_O_4_N_6_	7.53	477.2254	2.0	409.1617, 365.1720, 310.1296, 282.1354, 205.0731, 161.1074
M448	[-CH_2_]+[O]	C_24_H_28_O_3_N_6_	7.33–8.20	449.2312	3.6	363.1561, 337.1768, 308.1144, 282.1346, 161.1072
M462-1	[O]	C_25_H_30_O_3_N_6_	8.87	463.2471	4.1	379.1875, 324.1453, 296.1141, 201.0771, 161.1078
M464-1	[O]+[2H]	C_25_H_32_O_3_N_6_	9.53	465.2621	2.8	379.1875, 324.1451, 296.1144, 161.1078
M638-1	[O]+[GluA]	C_31_H_38_O_9_N_6_	9.83	639.2781	1.3	463.2460, 395.1830, 377.1721, 294.0976, 217.0718, 161.1075
M638-2	[O]+[GluA]	C_31_H_38_O_9_N_6_		639.2780	1.1	463.2457, 395.1827, 294.0999, 203.1290, 84.0816
M462-2	[O]	C_25_H_30_O_3_N_6_	10.03	463.2465	2.8	379.1874, 324.1458, 296.1136, 120.0810, 86.0971
M476-2	2[O]+[-2H]	C_25_H_28_O_4_N_6_		477.2255	2.2	409.1615, 365.1721, 310.1299, 282.1349, 205.0722, 161.1073
M462-3	[O]	C_25_H_30_O_3_N_6_	11.33-11.37	463.2467	3.3	395.1824, 377.1719, 322.1293, 294.0984, 217.0726, 120.0811
M542	[O]+[SO_3_]	C_25_H_30_O_6_N_6_S	12.53	543.2034	2.5	463.2452, 395.1829, 377.1718, 322.1296, 294.0986, 217.0718
M464-2	[O]+[-2H]	C_25_H_32_O_3_N_6_	13.87	465.2624	3.3	379.1876, 324.1459, 296.1145, 219.0884, 136.0757, 120.0810
M476-3	2[O]+[-2H]	C_25_H_28_O_4_N_6_	14.53	477.2264	3.4	393.1670, 322.1299, 120.0808

#### 3.4.1 Plasma

In AUC-pooled 0–24 h plasma, only SHR6390 was detected (Figure 3). The main reason was that the radioactivity in plasma was too low, and the signals of metabolites could not be distinguished from the background signal.

#### 3.4.2 Urine

In the 0–120 h pooled urine sample, a total of 6 radio-chromatographic peaks were identified, and the major peak was the parent SHR6390 (Figure 3), accounting for 14.11% of the dose. Five metabolites were assigned as M478, M448, M638-1/M638-2 (coeluting) and M462-3, accounting for 1.53%, 0.63%, 1.55% and 2.95% of the dose, respectively.

**FIGURE 4:**
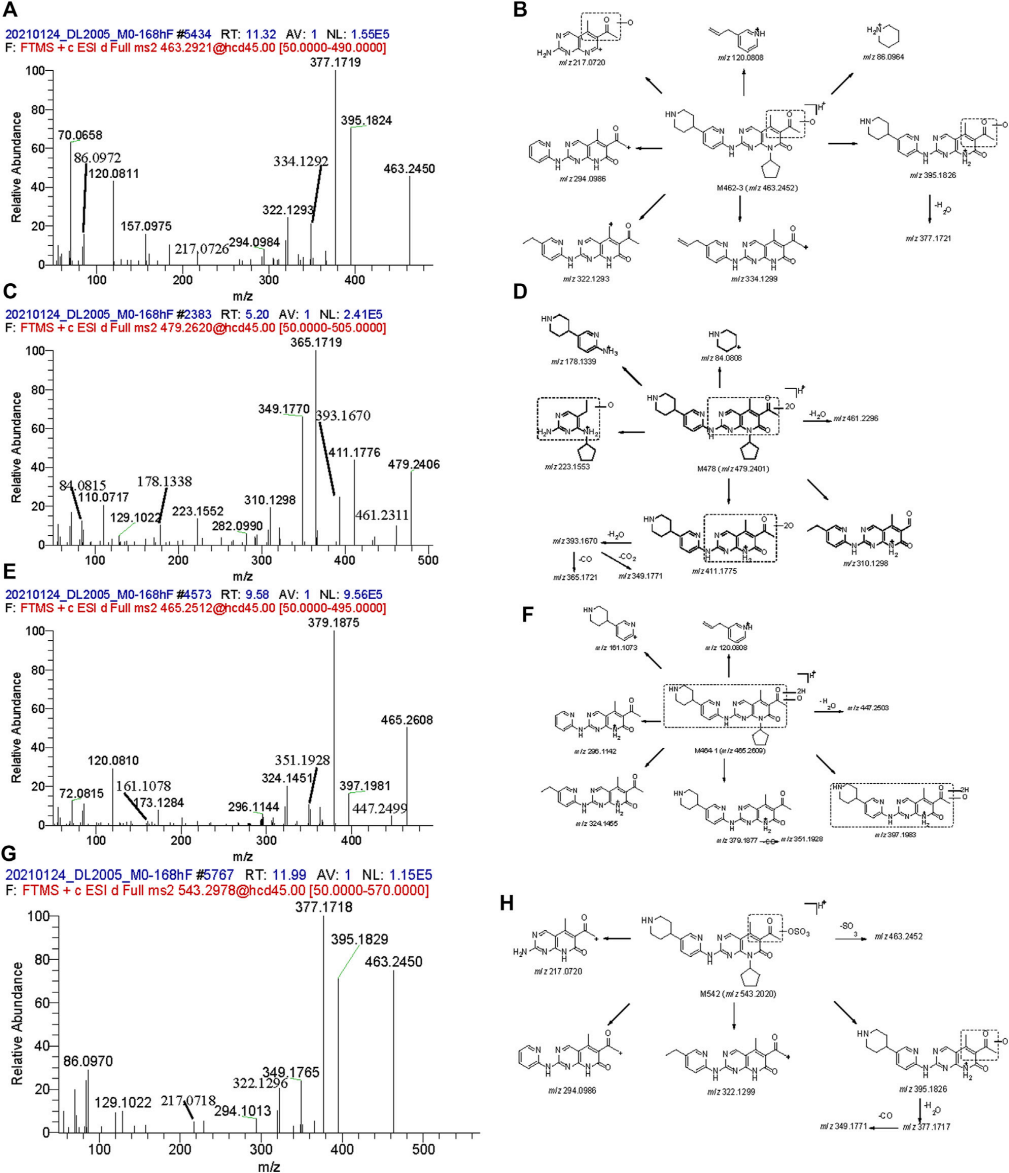
Mass spectra and the proposed fragmentation profiles of metabolites: M462-3 **(A, B)**, M478 **(C, D)**, M464-1 **(E, F)**, and M542 **(G, H)** in ESI (+).

M462-3: The signal of MS showed that the elemental change of M462-3 might be an oxygen atom more than SHR6390. The main fragment ions were *m/z* 120.0808, 217.0720, 294.0984, 322.1293, 334.1299, 377.1721 and 395.1826. Comparing the ion fragments with those of the parent, the structure of M464-1 was deduced, as shown in Figures 4A, B.

M478: The signal of MS showed that the elemental change of M478 might be di-oxidation of parent SHR6390. The main fragment ions were *m/z* 178.1339, 223.1553, 349.1770, 365.1721, 393.1670 and 411.1775. Comparing the ion fragments with those of the parent, the structure of M464-1 was deduced, as shown in Figures 4C, D.

In addition, M448 and two mono-oxidation and phase Ⅱ glucuronide acid conjugates (M638-1 and M638-2) were also detected as minor metabolites in urine.

#### 3.4.3 Stool

Parent SHR6390 and 12 metabolites were identified in the pooled 0–168 h fecal samples (Figure 3). Among them, SHR6390 was the predominant component (16%), and three abundant metabolites, M478, M464-1 and M542, accounted for 7.16%, 7.07% and 9.03% of the dose, respectively.

M464-1: The signal of MS showed that the elemental change of M478 was di-oxidation of parent SHR6390. The main product ions were *m/z* 161.1073, 296.1142, 324.1455 and 379.1877,397.1983. Comparing the ion fragments with those of the parent, the structure of M464-1 was deduced, as shown in Figures 4E, F.

M478: The details are shown in the urine section above.

M542: The signal of MS showed that the elemental change of M542 might be mono-oxidation and sulfation of the parent SHR6390. The main product ions were *m/z* 217.0720, 294.0986, 322.1299, 377.1717, 395.1926 and 463.2452. Comparing the ion fragments with those of the parent, the structure of M464-1 was deduced, as shown in Figures 4G, H.

In addition, M448, M462-1, M462-2, M462-3, M464-2, M476-1, M476-2 and M476-3 were also identified in stool as minor metabolites.

## 4 Discussion

This study reported the mass balance study of [^14^C]SHR6390 in human. After oral administration, 94.63% of the dosed radioactivity was recovered in urine and stool by 312 h post-dose, which indicated complete excretion, with 22.69% in urine and 71.93% in stool.

Based on high radioactivity recovery in sample extraction, the metabolite profiles were evaluated. A total of 13 metabolites were identified, and unchanged SHR6390 was the major metabolite in three matrix following an oral administration of [^14^C]SHR6390. In plasma, because of the low level of radioactivity, only SHR6390 was detected in pooled AUC_0-24h_ plasma. In urine and stool, SHR6390 was the major component; 5 and 12 metabolites were identified, respectively.

**FIGURE 5:**
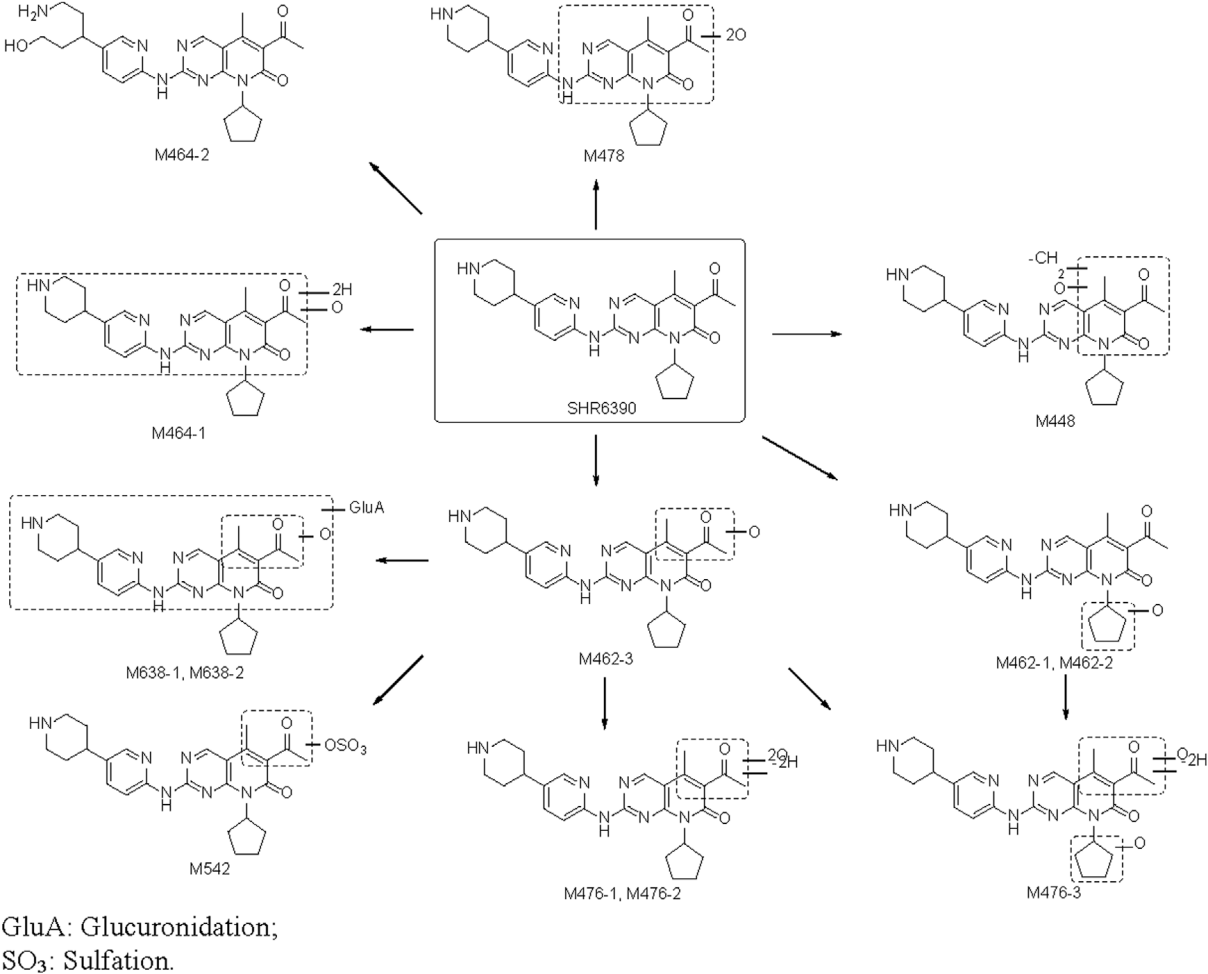
Metabolic pathway of SHR6390 in healthy Chinese male subjects.

The proposed biotransformation pathway based on findings from the present metabolism study is shown in Figure 5. The major metabolic pathways might be oxidation, glucuronidation and sulfation. As shown in Figure 5, the most susceptible metabolic spot of SHR6390 is the methyl on pyridyl pyrimidine and methyl of the acetyl group. The product ions at *m/z* 322.1293 and 294.0986 were used as diagnostic ions to determine the location of metabolism by summarizing the MS^2^ spectra of SHR6390 and the available reference standards for the main metabolites. This rule applies in most cases, with occasional exceptions.

In plasma, for the radioactivity concentration-time profiles, the mean *C*_max_ value of radioactivity was 166 ng eq/mL, and the mean AUC_last_ value was 1,670 ng eq./mL h. The mean *T*_max_ and *t*_1/2_ were approximately 3.17 and 17.50 h, respectively. The blood-to-plasma AUC_inf_ ratio (BPAR) of the radioactivity was 1.81. For the observed blood-to-plasma ratio, it should be detected and identification the metabolites in blood to determine a more complete metabolic profiling. For SHR6390 in plasma, the mean Cmax value of radioactivity was 42.9 ng/mL, and the mean AUC_0-t_ value was 1,150 ng/mL h. The mean AUC_0-inf_ value was 1,250 ng/mL h. The mean *T*_max_ and *t*_1/2_ were approximately 3.00 and 43.5 h, respectively.

Ribociclib was a medicine with similar structure. Concentrations of total radioactivity in blood and plasma were measured by AMS. The radioactivity mean *t*_1/2_ in plasma of ribociclib were was 293 h. The mean *C*_max_ value of radioactivity was 1,140 ng eq/mL. The mean AUC_0-inf_ value was 37,200 ng eq/mL*h (Alexander et al., 2020). The *C*_max_ and AUC_0-inf_ of SHR6390 was lower. SHR6390 may have better activity. The differences in data do not fully explain the superiority of drugs, and may also be caused by differences in detection methods.

In conclusion, this study shows that after a single oral administration of [^14^C] SHR6390, 94.63% of the dose was recovered in urine and feces, of which 22.69% was recovered in urine and 71.93% in feces. The characterization of SHR6390 pharmacokinetics, mass balance and metabolism is helpful to guide our understanding of SHR6290 metabolism and elimination pathway. In plasma, for the low level of radioactivity, only SHR6390 was detected in pooled AUC_0–24 h_ plasma. In urine and stool, SHR6390 was the major component; 5 and 12 metabolites were identified, respectively. Overall, faecal elimination played a significant role.

## Data Availability

The original contributions presented in the study are included in the article/supplementary material, further inquiries can be directed to the corresponding authors.
